# Interventions to improve well-being among children and youth aged 6–17 years during the COVID-19 pandemic: a systematic review

**DOI:** 10.1186/s12916-023-02828-4

**Published:** 2023-04-03

**Authors:** Stephana J. Moss, Sara J. Mizen, Maia Stelfox, Rebecca Brundin Mather, Emily A. FitzGerald, Perri Tutelman, Nicole Racine, Kathryn A. Birnie, Kirsten M. Fiest, Henry T. Stelfox, Jeanna Parsons Leigh

**Affiliations:** 1 Faculty of Health, School of Health Administration, Dalhousie University, Halifax, NS Canada; 2 Cumming School of Medicine, Department of Critical Care Medicine, University of Calgary, Calgary, AB Canada; 3 Faculty of Science, Department of Psychology and Neuroscience, Dalhousie University, Halifax, NS Canada; 4 Faculty of Social Sciences, School of Psychology, University of Ottawa, Ottawa, ON Canada; 5 Cumming School of Medicine, Department of Anesthesiology, Perioperative and Pain Medicine, University of Calgary, Alberta, Canada; 6 Cumming School of Medicine, Department of Community Health Sciences, Calgary, AB Canada; 7 O’Brien Institute for Public Health, University of Calgary, Calgary, AB Canada

**Keywords:** Child, Youth, COVID-19, Well-being, Intervention, Systematic review

## Abstract

**Background:**

The COVID-19 pandemic is an example of a global infectious disease outbreak that poses a threat to the well-being of children and youth (e.g., physical infection, psychological impacts). The consequences of challenges faced during COVID-19 may be longstanding and newly developed interventions are being deployed. We present a narrative synthesis of available evidence from the first 2 years of the COVID-19 pandemic on the feasibility, accessibility, and effects of interventions to improve well-being among children and youth to inform the development and refinement of interventions relevant to post-pandemic recovery.

**Methods:**

Six databases were searched from inception to August 2022. A total of 5484 records were screened, 39 were reviewed in full text, and 19 studies were included. The definition of well-being and the five domains of well-being as defined by the Partnership for Maternal, Newborn & Child Health and the World Health Organization in collaboration with the United Nations H6 + Technical Working Group on Adolescent Health and Well-Being were used.

**Results:**

Nineteen studies (74% randomized controlled trials) from 10 countries were identified, involving a total of 7492 children and youth (age range: 8.2–17.2 years; 27.8–75.2% males) and 954 parents that occurred during the COVID-19 pandemic (March 2020 to March 2021). Nearly all interventions (*n* = 18, 95%) targeted health and nutrition, followed by connectedness (*n* = 6, 32%), while fewer studies targeted agency and resilience (*n* = 5, 23%), learning and competence (*n* = 2, 11%), or safety and support (*n* = 1, 3%). Five interventions (26%) were self-guided while 13 interventions (68%) were guided synchronous by a trained professional, all of which targeted physical and mental health subdomains within health and nutrition; one intervention (5%) was unclear.

**Conclusions:**

Studies deploying synchronous interventions most often reported improved well-being among children and youth largely in the domain of health and nutrition, specifically physical and mental health. Targeted approaches will be crucial to reach sub-groups of children and youth who are most at risk of negative well-being outcomes. Further research is needed to determine how interventions that best supported children and youth early in the pandemic are different from interventions that are required now as we enter into the post-pandemic phase.

**Supplementary Information:**

The online version contains supplementary material available at 10.1186/s12916-023-02828-4.

## Background

Since the onset of the COVID-19 pandemic, rigorous efforts have been made by governments and public health officials worldwide to protect the public from disease transmission, including widespread closure of public institutions, implementation of mandatory masking and physical distancing policies, travel restrictions, and “stay at home” orders [1]. Evidence and theory suggest that the COVID-19 pandemic and associated public health measures have taken a devastating toll on children and youth through a number of mechanisms [2, 3]. Social isolation resulting from these measures as well as reduction in social contacts between children and youth sequesters children and youth and reduces opportunities for cognitive and social development [4, 5]. This may also increase anxiety due to the loss of familiar and cherished activities and the absence of the protective effects of connection with school [6]. Combined with fewer opportunities to engage in protective behaviors such as physical activity, public health measures enacted because of the COVID-19 pandemic are likely to have detrimental short- and long-term effects on youth mental well-being [7], particularly among youth with pre-existing vulnerabilities such as familial adversity [8].

The Partnership for Maternal, Newborn & Child Health, and the World Health Organization (WHO) led an initiative of the United Nations H6 + Technical Working Group on Adolescent Health and Well-Being to develop a consensus framework for defining, programming, and measuring adolescent well-being, ultimately defined as that “children and youth have the support, confidence, and resources to thrive in contexts of secure and healthy relationships, realizing their full potential and rights,” including five domains (i.e., health and nutrition, learning and competence, connectedness, safety and support, and agency and resilience) [9]. Child and youth well-being is conceptualized differently for every individual, group, and community that is dependent on culture and values [10]. To “be well” requires that conditions are in place to allow children and youth to reach their full potential [11]. Well-being is shaped by the quality of a person’s experiences, which are, in turn, influenced by a number of factors from familial to societal [12, 13]. Adverse early life experiences can have long-lasting effects across the entire life course, enduring inequalities and resulting in damaging consequences for the health and well-being of both individuals and society [14, 15]. The COVID-19 pandemic can be conceptualized as early life adversity, and COVID-19-perceived stressors may be a key mechanism underlying the development of adverse psychosocial outcomes [16]. Children and youth with pre-existing mental health problems may be disproportionately affected by the traumatic effects of the COVID-19 pandemic [17].

Existing literature reviews on children and youth in the context of the COVID-19 pandemic have focused primarily on mental health outcomes [18, 19], often characterizing the impact of specific public health measures (e.g., school closures) [20, 21]. International reports have highlighted potentially important interventions to protect children and youth from the possible wide-ranging well-being harms associated with the COVID-19 pandemic [22, 23]. Interventions that best supported the needs of children and youth early in the pandemic are likely different than those needed during the post-pandemic phase [24]; systematically evaluating the efficacy of the existing interventions is crucial to understanding how to support children and youth that remains a relevant and urgent issue. There is very limited knowledge on the impact of interventions especially among priority sub-populations (e.g., low socioeconomic status (SES)) of children and youth, expected to be most at risk of poor well-being outcomes as a consequence of the COVID-19 pandemic [25]. We present here a narrative synthesis summarizing the available evidence from the first 2 years of the COVID-19 pandemic on the feasibility, accessibility, and effects of interventions designed to improve well-being among children and youth.

## Methods

We undertook a systematic review and narrative synthesis to answer: What are the effects of interventions deployed within the COVID-19 pandemic to improve well-being among children and youth? We followed the relevant requirements of the Preferred Reporting Items for Systematic Reviews and Meta-Analyses (PRISMA) reporting guidelines (Additional file 1: Table S1) [26] and the Synthesis Without Meta-analyses (SWiM) reporting guidelines (Additional file 1: Table S2) [27], and our protocol was prospectively registered with PROSPERO (CRD42022307248). No changes to the protocol were applied. For this review, we focused on randomized controlled trials (RCTs) and cluster randomized controlled trials (CRTs) to provide the highest quality evidence to facilitate the effective development of future interventions.

The components of population, intervention, comparator, outcome, study design, and timeframe are as follows:

- *Population*: any child or youth aged 6 to 17 years
- *Intervention*: Any intervention targeted to improve at least one of five domains (i.e., health and nutrition, learning and competence, connectedness, safety and support, and agency and resilience) of child and youth well-being [9]
- *Comparator*: any comparator
- *Outcomes*: any outcome within the five domains of child and youth well-being
- *Study design*: randomized controlled trial or cluster randomized trial
- *Timeframe*: publications from 01 December 2019 to 01 August 2022

### Search strategy

Search strategies were developed and reviewed by a medical librarian (DLL). We searched six databases (MEDLINE, Embase, Cinahl, PsycInfo, Healthstar, and Cochrane Trials Database) from inception to August 1, 2022 (for greater inclusivity with regard to captured studies to screen subsequently within our pre-specified timeline). We used a combination of free-text controlled items to identify citations containing children and youth and concepts of and related to well-being. We employed a well-known existing search strategy for RCTs that has 93.8% sensitivity for CRTs. The search strategy for MEDLINE is available in Additional file 1: Table S3. We screened the reference list of included articles and asked experts in the field for additional studies.

For the purposes of this review, we adopted the following definition for well-being as defined by the Partnership for Maternal, Newborn & Child Health, and the WHO in collaboration with the United Nations H6 + Technical Working Group on Adolescent Health and Well-Being: “children and youth have the support, confidence, and resources to thrive in contexts of secure and healthy relationships, realizing their full potential and rights,” and the five well-being domains provided in the published framework (i.e., health and nutrition, learning and competence, connectedness, safety and support, and agency and resilience) (Table 1) [9]. The inclusion criteria included any children and youth aged 6 up to 18 years, capturing the portion of children and youth in adolescence and emerging independence [28, 29] who perceived large, socio-emotional impacts of the COVID-19 pandemic [30, 31], and an intervention targeted to improve at least one of the five aforementioned well-being domains [9]. Studies reporting pharmacological interventions were eligible for inclusion. Studies did not need to report on family member outcomes though these studies were included if they did; both self-report and proxy-report outcomes were accepted as eligible. Interventional studies with all study designs were included. Pre-print records and conference abstracts were excluded to omit studies that were not yet peer-reviewed. No language restrictions were applied to our database search nor to the inclusion of articles.

**Table 1:** Classification framework for well-being domains and subdomains^a^

Domain	Subdomains
*(1) Good health and optimum nutrition*	• Physical health and capacities • Mental health and capacities • Optimal nutritional status and diet
*(2) Connectedness, positive values, and contribution to society*	• Connectedness: is part of positive social and cultural networks and has positive, meaningful relationships with others, including family, peers, and, where relevant, teachers and employers • Valued and respected by others and accepted as part of the community • Attitudes: responsible, caring, and has respect for others; has a sense of ethics, integrity, and morality • Interpersonal skills: empathy, friendship skills, and sensitivity • Activity: socially, culturally, and civically active • Change and development: equipped to contribute to change and development in their own lives and/or in their communities
*(3) Safety and a supportive environment*	• Safety: emotional and physical safety • Material conditions in the physical environment are met • Equity: treated fairly and have an equal chance in life • Equality: equal distribution of power, resources, rights, and opportunities for all • Nondiscrimination • Privacy • Responsive: enriching the opportunities available to the adolescent
*(4) Learning, competence, education, skills, and employability*	• Learning: has the commitment to, and motivation for, continual learning • Education • Resources, life skills, and competencies: has the necessary cognitive, social, creative, and emotional resources; skills (life/decision-making); and competencies to thrive, including knowing their rights and how to claim them, and how to plan and make choices • Skills: acquisition of technical, vocational, business, and creative skills to be able to take advantage of current or future economic, cultural, and social opportunities • Employability • Confidence that they can do things well
*(5) Agency and resilience*	• Agency: has self-esteem; a sense of agency and of being empowered to make meaningful choices and to influence their social, political, and material environment; and capacity for self-expression and self-direction appropriate to their evolving capacities and stage of development • Identity: feels comfortable in their own self and with their identity(s), including their physical, cultural, social, sexual, and gender identity • Purpose: has a sense of purpose, desire to succeed, and optimism about the future • Resilience: equipped to handle adversities both now and in the future, in a way that is appropriate to their evolving capacities and stage of development • Fulfilment: feels that they are fulfilling their potential now and that they will be able to do so in the future

^a^Ross DA, Hinton R, Melles-Brewer M, et al. Adolescent well-being: a definition and conceptual framework. J Adolesc Health. 2020;67(4):472–476

We initially sought to identify data on interventions to improve well-being that were deployed before the COVID-19 pandemic (in periods of similar health crises) as well as during the COVID-19 pandemic. Given that interventions during the COVID-19 pandemic very frequently occurred within the context of broader social lockdowns, our initial search strategy was broad to capture a range of outcomes in the contexts of interventions with and without co-occurring lockdowns. The available literature from before COVID-19 in times of similar health crises was sparse and was therefore not included in this review. Additional identified data from non-interventional studies on the association of strategies or approaches to improve child and youth well-being will be published elsewhere.

After a subset of the team (SJM1, SJM2, EF, RBM) achieved 100% agreement on a pilot test of 50 random citations, all titles and abstracts were reviewed independently in duplicate by two reviewers (SJM1; and any of SJM2, EF, or RBM). Any study selected by any reviewer at this stage progressed to the next stage. The full text of all articles was reviewed independently in duplicate by two reviewers (SJM1; and any of SJM2, EF, or RBM); articles selected by both reviewers at this stage were included in the final review. Disagreements were resolved by discussion and or the involvement of a third reviewer when necessary. References were managed in Endnote X9 (Clarivate Analytics, Philadelphia, USA).

### Data extraction and risk of bias assessment

After another calibration exercise to achieve 100% agreement, two authors (SJM1; and any of SJM2, EF, or RBM) independently and in duplicate extracted outcome data that were verified independently by 1 author (MS). Information on document characteristics (e.g., year of publication, geographic location), study characteristics (e.g., setting, sites), youth and family characteristics (e.g., age, gender, relationship), intervention characteristics (e.g., type, components), well-being outcomes (i.e., health and nutrition, learning and competence, connectedness, safety and support, and agency and resilience of child and youth well-being [9]), statistical significance (e.g., *p*-values, measures of variance), and authors’ conclusions were collected. Included studies with notable subgroups determined from the study eligibility criteria (e.g., youth with developmental disorders) were recorded. Studies that assessed parental well-being outcomes as a secondary outcome were noted. Evidence was assessed for risk of bias by using the Adapted Cochrane Risk of Bias Assessment Tool for assessing the risk of bias for all study designs [32].

### Data synthesis and analysis

Owing to the heterogeneity of interventions and study designs, including small samples, meta-analysis was not possible. We performed a narrative synthesis of the results, grouping studies according to the type of well-being domain [9]. The World Bank’s Classification of Countries by income was applied to categorize included countries by income type. For multi-arm trials, we contacted the primary author to obtain data among all experimental groups if not reported. For cluster-crossover trials, we contacted the primary author to obtain data accounting for the opposite effects of the variance of clustering and crossover. For piloting studies that grew to larger (e.g., evaluative) studies, we contacted the primary author to ensure uniqueness in data sets to prevent duplication of data. All relevant outcomes within the included studies are reported, and data reported is as published in the included manuscript or as provided by the primary author; no standardization metric or transformation methods were applied. We considered two-sided *p* < 0.05 as statistically significant. We did not conduct a formal quantitative or qualitative assessment of reporting bias or publication bias.

## Results

**Fig. 1:**
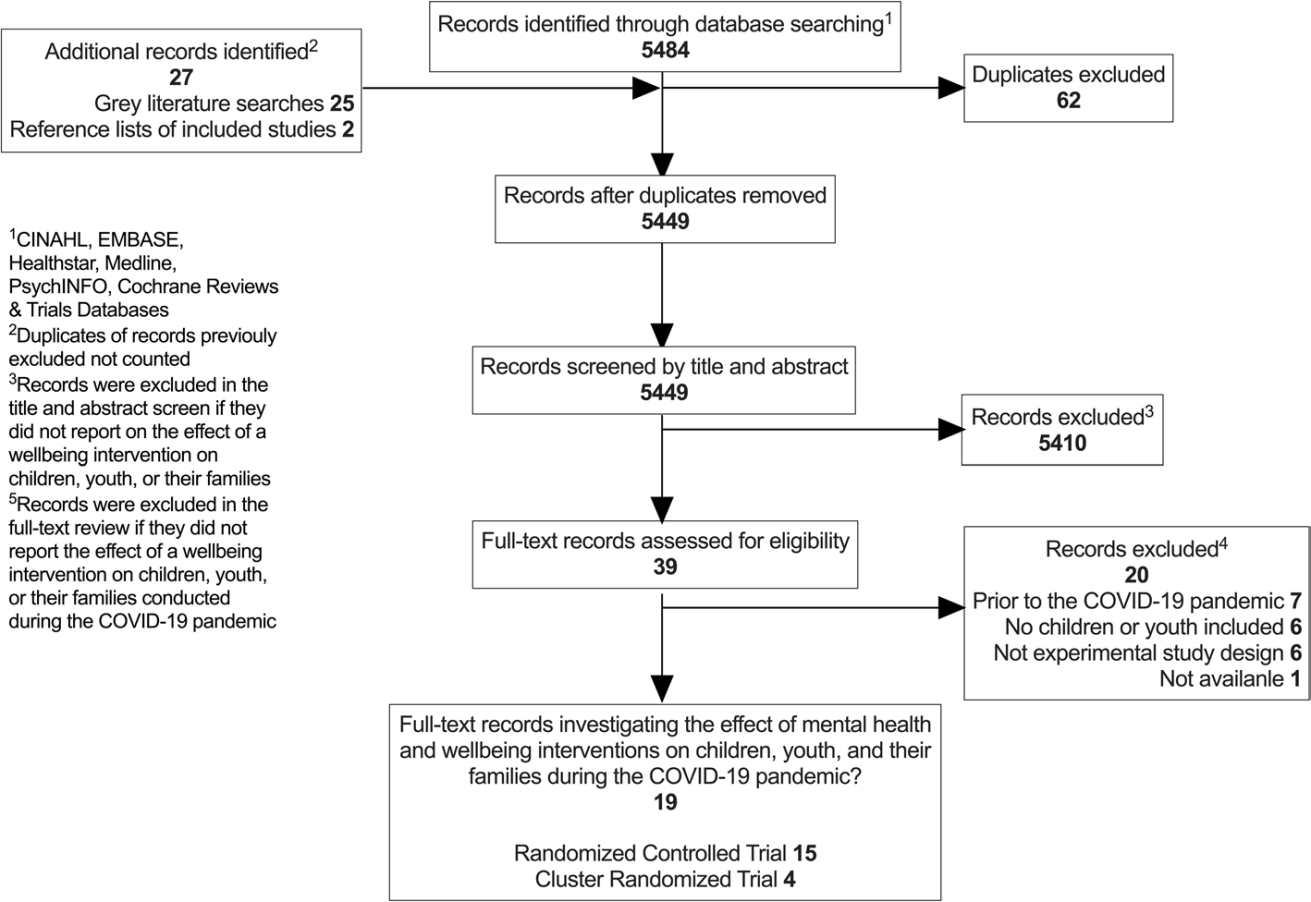
Study flow chart

Figure 1 shows the search flow. A total of 5449 records were retrieved after removing duplicates; 39 were reviewed in full text. Here, we report findings from 19 studies on well-being interventions for children and youth (Table 2) [33–51]. Studies involved a total of 7492 children and youth and 954 parents, all of which occurred during the first year of the COVID-19 pandemic (March 2020 to March 2021).

**Table 2:** Characteristics of included studies organized alphabetically by source

Source	Country	Design^1^	Site (*N*)	Setting^2^	Intervention	Duration^3^	Follow-up	Child/ youth (*N*)	Family (*N*)	Child/ youth age (mean)^4^	Child/ youth SES^5^
Cataldi	Italy	RCT	1	Research clinic	CrossFit program	8 weeks	2 months	30	N/A	17.2 (0.5)	N/R
Chen*	China	RCT	5	Schools	Mindfulness training and aerobic exercise	8 weeks	1 month	2120	N/A	14.4 (1.0)	N/R
Choi	Hong Kong	CRT	1	Research clinic	Optical defocus treatment	18–24 months	None	115	N/A	10.3 (1.5)	N/R
Cruwys	Australia	RCT	N/R	N/A	Cognitive behavior therapy and Groups 4 Health	8 weeks	12 months	174	N/A	19 (2.0)	N/R
Ding*	China	RCT	N/A	Convenience	Peer education	8 weeks	None	150	N/A	15.3 (2.3)	N/R
Gadari*	Iran	RCT	2	Schools	Resilience training	6 weeks	1 month	80	N/A	Between 9 and 10 years	N/R
Gulesci	Bolivia	RCT	N/A	Convenience	Empowerment program	3 months	7 months	600	N/A	Between 15 and 18 years	Low
Lee*	Korea	RCT	N/R	N/A	Physical education class	10 weeks	1.5 months	54	N/A	15.9 (0.4)	N/R
Liu*	China	RCT	3	Communities	Logotherapy-based mindfulness	8 weeks	None	121	N/A	15.3 (0.8)	Middle
Malboeuf-Hurtubise (a)*	Canada	CRT	2	Classrooms	Art therapy experientials	5 weeks	1 week	22	N/A	11.3	N/R
Malboeuf-Hurtubise (b)*	Canada	CRT	5	Classrooms	Philosophy for children	5 weeks	1 week	37	N/A	8.2	N/R
Mesurado*	Argentina	CRT	10	Courses	Virtual Hero Program	7 weeks	1 week	211	N/A	14.1 (1.1)	N/R
Sarti*	Italy	RCT	N/A	Convenience	Telerehabilitation	3–4 months	None	56	N/A	10.8	N/R
Schleider*	USA	RCT	N/A	Convenience	Single-session interventions	20–30 min	3 months	2452	N/A	Between 13 and 16 years	N/R
Shao	China	RCT	4	Communities	Dance therapy	7–8 weeks	None	62	NA	15.8 (0.6)	N/R
Yadav*	USA	RCT	N/A	Convenience	Heartfulness meditation and brainwave entrainment	4 weeks	None	40	NA	Between 14 and 18 years	N/R
Zhang	China	RCT	3	Communities	Psychological counselling	8 weeks	None	153	NA	15.8 (1.5)	Mixed
Zheng*	China	CRT	12	Schools	Peer-to-peer support	N/R	2 weeks	954	954	13.5 (0.5)	Mixed
Zuo*	China	RCT	1	Schools	Divergent thinking training	9 days	None	61	NA	N/R	N/R

^1^*CRT*, cluster randomized trial; *RCT*, randomized controlled trial

^2^Convenience sample indicates online recruitment (e.g., e-mailing lists, social media)

^3^Total duration of intervention

^4^Standard deviation provided in round brackets if mean reported in manuscript

^5^Socioeconomic status (SES) of the majority of the cohort

^*^Indicates part or full intervention delivered online or via social medica application

Fifteen studies (79%) were person-level RCTs [34, 35, 41, 49, 51], and four studies (21%) were cluster RCTs [42–44, 50]. Four studies (21%) deployed interventions previously validated in prior work. A single study (5%) performed a needs assessment among children and youth and engaged children and youth in the development and refinement of the intervention. Five interventions (26%) were self-guided while 13 interventions (68%) were guided by a trained professional; one intervention (5%) was unclear. No study reported a pharmacological intervention. Four studies (21%) reported on the socioeconomic diversity of the children or youth participants; two studies (11%) focused specifically on low socioeconomic children and youth participants, and two studies (11%) enrolled children or youth participants with broad socioeconomic diversity. Eight studies (42%) were from low- and middle-income countries (seven from China and one from Iran); two studies (11%) each were from Canada, the USA, or Italy; one study (5%) each was from Argentina, Bolivia, Korea, Ireland, Australia, or Hong Kong. All studies were published in English.

**Fig. 2:**
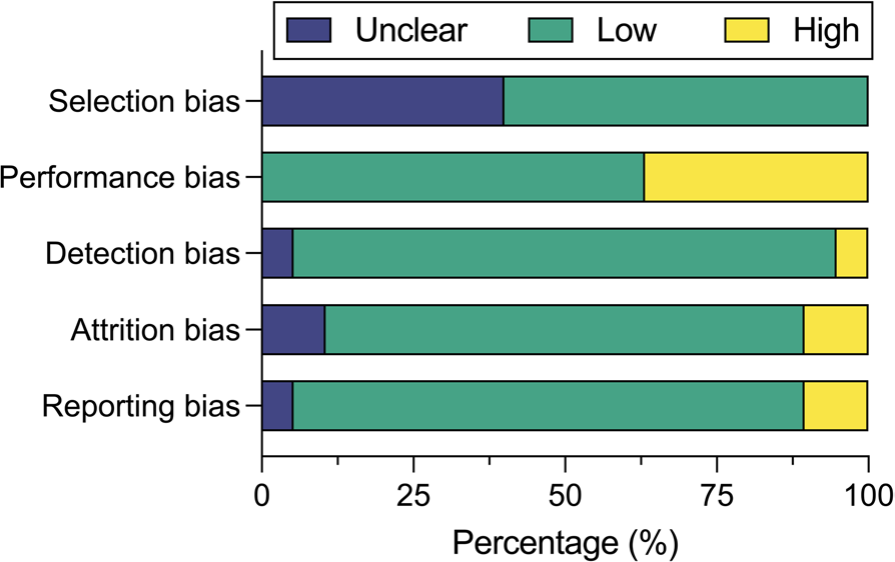
Risk of bias assessment for included studies

Nine studies (47%) had a low risk for bias, and eight studies (42%) had a high risk for bias, while two studies (11%) were unclear (Fig. 2; Additional file 1: Table S4). Among the fourteen site-based interventions, 12 studies (63%) reported on the number of sites at which the intervention was deployed; four studies (21%) used school settings, three studies (16%) used community settings, two studies (11%) used classrooms, two studies (11%) used research clinic settings, and one study (5%) used a course to deploy the intervention; two studies (11%) did not report on the site(s) of intervention deployment. Four studies (21%) were online interventions that used convenience sampling to recruit participants from several regions in their respective countries. The majority of studies (*n* = 17, 89%) assessed the intervention outcomes using validated assessment tools, five (26%) of which also included single-response questions; two studies (11%) assessed anthropometrics at follow-up. Seventeen studies (89%) identified positive outcomes to improve well-being after completion of the intervention, and two studies (11%) identified neither positive nor negative outcomes, such as adverse events, harm, or distress associated with the intervention. A summary of findings from interventions by the last point of outcome assessment is provided in Table 3. Additional file 2 provides all reported data across time points from included studies.

**Table 3:** Summary of findings from interventions by last point of outcome assessment

Source (design)	Intervention domain(s)^1^	Last time of assessment	Outcome measure(s)	Significant effect of intervention^2^
**Immediately post-intervention**				
Choi (CRT)	Health and nutrition	N/A	Myopia progression	Slowed myopia progression
Ding (RCT)	Health and nutrition	N/A	Anxiety; depression; sleep quality	Decreased anxiety; decreased depression; increased sleep quality
Liu (RCT)	Health and nutrition Agency and resilience	N/A	Anxiety; depression; Internet addiction; negative coping; positive coping	Decreased anxiety; decreased depression; decreased Internet addiction; decreased negative coping; increased positive coping
Sarti (RCT)	Health and nutrition Connectedness Learning and competence	N/A	Learning; negative emotions; positive emotions; well-being	Improved learning; increased positive emotions; increased well-being
Shao (RCT)	Health and nutrition Agency and resilience	N/A	Anxiety; depression; satisfaction; resilience	Decreased anxiety; decreased depression; increased satisfaction; increased resilience
Yadav (RCT)	Health and nutrition Learning and competence	N/A	Anger; attention; depression; memory; mood; stress; sleep quality	Decreased anger; improved mood; decreased stress; improved sleep
Zhang (RCT)	Health and nutrition Agency and resilience	N/A	Anxiety; depression; resilience	Decreased anxiety; decreased depression; increased resilience
Zuo (RCT)	Health and nutrition	N/A	Anxiety; depression; Self-efficacy	Decreased anxiety; increased self-efficacy
**Intervention follow-up**				
Malboeuf-Hurtubise (a) (CRT)	Health and nutrition Connectedness	1 week	Anxiety; depression; hyperactivity; inattention	Decreased hyperactivity; decreased inattention
Malboeuf-Hurtubise (b) (CRT)	Health and nutrition Connectedness	1 week	Anxiety; inattention; satisfaction	Decreased anxiety; decreased depression; increased satisfaction
Mesurado (CRT)	Connectedness	1 week	Kindness and generosity; positive emotions	Increased kindness and generosity; increased positive emotions
Zheng (CRT)	Health and nutrition	2 weeks	Anxiety; eye strain; sleep quality	Decrease anxiety; decreased eye strain
Gadari (RCT)	Agency and resilience	1 month	Social self-efficacy	Increased self-efficacy
Chen (RCT)	Health and nutrition Connectedness	1 month	Anxiety; positive and negative affect; well-being	Decreased anxiety; increased positive affect; decreased negative affect
Lee (RCT)	Health and nutrition	1.5 months	Balance; muscular strength; fitness; flexibility	Increased balance; increased muscular strength; increased fitness; increased flexibility
Cataldi (RCT)	Health and nutrition	2 months	Anthropometrics; fitness; self-efficacy	Improved anthropometrics; increased fitness; increased self-efficacy
Schleider (RCT)	Health and nutrition Connectedness Agency and resilience	3 months	Agency; anxiety; cognition; depression; hopelessness; fatigue; trauma; restricted eating	Increased agency; decreased depression; decreased hopelessness; decreased restrictive eating
Gulesci (RCT)	Health and nutrition Safety and support	7 months	Income; soft skills; violence	Increased income; decreased violence
Cruwys (RCT)	Health and nutrition	12 months	Depression; loneliness; well-being	Decreased depression; decreased loneliness; increased well-being

^1^Well-being domain according to Ross et al.’s *Adolescent well-being: a definition and conceptual framework*

^2^Effect deemed significant when at least *p* < 0.05 in comparison with control

The exposure in all studies was public health measures mandated during the COVID-19 pandemic. School closures were what impacted children and youth the most within the first years of the pandemic. Only one study investigated a single exposure that increased screen time because of the transition from in-person to online. Descriptions of all included studies are provided subsequently.

### Health and nutrition domain

All but three studies [38, 39, 44] assessed the effect of an intervention on the health and nutrition of 7201 collective children and youth during the COVID-19 pandemic; this included 12 person-level RCTs, three cluster RCTs, and one quasi-experimental study. Reported findings included slowed myopia progression [35]; decreased anxiety and depression with increased sleep quality [37]; decreased anxiety, depression, and Internet addiction [41]; improved learning [45]; decreased anxiety and depression with increased life satisfaction [47]; decreased anger and improved mood [48]; decreased anxiety and depression [49]; decreased anxiety with increased self-efficacy [51], decreased hyperactivity [43]; decreased anxiety and depression [42]; decreased anxiety [34, 50]; increased balance, muscular strength, fitness, and flexibility [40]; improved anthropometrics with increased fitness and self-efficacy [33]; increased agency with decreased depression [46]; increased income [39]; and decreased depression and loneliness with increased well-being [36].

The following interventions comprised diverse components to target other well-being domains; only their findings on health and nutrition will be presented here with more detailed descriptions presented below. Chen and colleague’s mindfulness meditation and aerobic exercise RCT resulted in decreased anxiety (on the Self-Rating Anxiety Scale) at 1-month follow-up [34] while the Philosophy for Children intervention and Mindfulness-Based Intervention conducted by Malboeuf-Hurtubise and colleagues improved anxiety symptoms (on the Behavior Assessment Scale for Children-3rd edition) for children at 1-week follow-up [43]; no decrease in anxiety nor depression symptoms were observed by the art therapy experiential interventions described in another included study [43]. The three-group telerehabilitation program conducted by Sarti and colleagues revealed that children with specific learning disorders and cerebral palsy scored higher than typically developing children in the Respect Scale (of the Comprehensive Inventory for Thriving Children) of well-being experience [45]. Compared to controls, the SSI Intervention deployed by Schlieder and colleagues reduced 3-month depressive symptoms (on the Children’s Depression Inventory) and 3-month restrictive eating (measured using the Dietary Restriction Screener), while the Heartfulness Meditation and Brainwave Entrainment interventions by Yadav and colleagues decreased anger, improved mood, and decreased stress (on the Perceived Stress Scale, Patient Health Question-9, and the Profile of Mood States), but only for adolescents who received the 4-week Heartfulness Meditation intervention [48]. Their results confirm the utility of free-of-charge, online SSIs for adolescents with elevated symptoms, even in the high-stress COVID-19 context.

Choi and colleagues compared the performance of defocus incorporated multiple segments lens with that of single vision lens treatment in reducing myopia progression that was found to be worsening during the COVID-19 pandemic [35]. In this exploratory analysis including 115 school children (mean age, 10.3 years; 49.5% males), they found that myopia progressed more rapidly in school children during the period when there were more COVID-19-related lockdown measures and that optical treatment with defocus incorporated multiple segments was significantly associated with slower myopia progression compared with single vision lens treatment during the COVID-19 lockdown period.

Ding and colleagues conducted a self-guided 8-week model 328-based peer education peer-education RCT with 150 youth (mean age, 15.3 years; 52.7% males) recruited through convenience on youth anxiety, depression, and sleep quality [37]. They found that their intervention significantly reduced anxiety and depression (via the Self-rating Anxiety and Depression scales) and increased sleep quality (measured using the Pittsburgh Sleep Quality Index).

An 8-week logotherapy-based mindfulness RCT was conducted by Liu and colleagues with the aim to improve internet addiction among 121 youth (mean age, 15.3 years; 75.2% males) in China. The authors did not specify how the intervention was guided. Their intervention not only improved Internet addiction (on the Internet Addiction Scale) and its five dimensions, but also improved both (self-rated) anxiety and depression.

Shao and colleagues investigated the intervention effect of professionally guided dance therapy based on the Satir model on the mental health of 62 adolescents (mean age, 15.7 years; 48% males) with depression located within 4 communities in China [47]. Compared to individual baseline assessments and control assessments, their intervention improved anxiety, depression (on the Anxiety and Depression Scale), and life satisfaction (measured using the Life Satisfaction Scale).

Zhang and colleagues explored the intervention effect of an 8-week research-based professionally guided psychological counseling program in combination with physical exercise on 152 adolescents’ mental health (mean age, 15.8 years; 54% males) [49]. They reported that the intervention improved anxiety and depression (on the Self-rating Anxiety and Depression Scales).

Zuo and colleagues conducted an RCT in one school in China on the effect of self-guided Divergent Thinking Training on teenager’s self-efficacy and emotions measured using the General Self-Efficacy Scale [51]. Participants in the experimental group were given a 9-day theme of “writing down 10 novel functions of the mask,” while those in the control group spent 10 min each day recording what they ate. No demographic data of participants were reported. Their results showed that for the experimental group compared to the control group, self-efficacy ceased decreasing while anxiety decreased for the experimental group.

The results from the Peer-to-Peer support intervention (including health education information promoting exercise and ocular relaxation, and access to a digital behavior change intervention, with live streaming and peer sharing of promoted activities) provided by Zheng and colleagues of grade 7 students across 12 schools in China in a cluster RCT indicated that their digital behavior change intervention reduced children’s anxiety (on the Spence Children’s Anxiety Scale) and eye strain (on the Computer Vision Syndrome Questionnaire), but not sleep quality (on the Pittsburgh Sleep Quality Index), during COVID-19-associated online schooling [50].

Lee and colleagues conducted an RCT in Korea to investigate the effects of 10 weeks of 2 times per week online physical education classes, using Tabata training, on 54 middle school students’ physical fitness (mean age, 15.9 years) [40]. Their results showed that the online physical education class had a positive effect on the improvement of muscle mass, ankle strength (dorsiflexion), hip strength (abduction, flexion, extension, and external rotation), knee strength (extension and flexion), and balance (Y-balance test) in adolescents.

The effectiveness of a professionally guided CrossFit program to mitigate the deficits in fitness caused by COVID-19 prevention measures and to evaluate the effects on self-efficacy in a group of 30 children and youth (mean age, 17.3 years; 60% males) was studied by Cataldi and colleagues [33]. Their intervention improved all fitness tests, as well as self-efficacy (on the Regulatory Emotional Self-efficacy Scale), with the authors concluding that their CrossFit intervention program could positively affect the general physical well-being and improve the emotional perceived self-efficacy in healthy adolescents.

Cruwys and colleagues performed a professional 8-week cognitive behavior therapy (CBT) and Groups 4 Health RCT performed on 174 healthy adolescents in Australia (mean age, 19.0 years; 27.8% males) [36]. The authors found that at 1-year follow-up, both CBT and Groups 4 Health led to symptom improvement, though the benefits of Groups 4 Health were more for depression, loneliness, and general well-being (on Short Warwick Edinburgh Mental Well-being Scale).

### Connectedness domain

Three complex cluster RCTs and three complex person-level RCTs focused on improving the connectedness of children and youth during the COVID-19 pandemic. These six included studies reported increased well-being [45]; increased positive affect and decreased negative affect [34]; decreased hopelessness [46]; increased kindness, generosity, and positive emotions [44]; decreased inattention [42]; and increased satisfaction [43].

The intervention deployed by Sarti and colleagues (described above) resulted in children with specific learning disorders and cerebral palsy scoring higher than typically developing children in Support and in Respect scales of the Comprehensive Inventory of Thriving Children [45].

Chen and colleagues explored the intervention effect of an 8-week meditation mindfulness training and aerobic exercise intervention on negative emotions as measured by the Positive and Negative Affect Scale of 2120 adolescents (mean age, 14.4 years; 51% males) with moderate or severe anxiety in five middle schools during the COVID-19 pandemic [34]. After the professionally guided intervention, the positive emotion score of the experiment group was higher than that of the control group, and the negative emotion score of the former was lower than that of the latter. The variances in the positive and negative emotion scores were higher in the experiment group than in the control group. The variance in the overall well-being index was also greater in the experiment group than in the control group. The authors concluded that their intervention has the potential to significantly increase positive emotions, decrease negative emotions, and improve the overall well-being of adolescents during the COVID-19 pandemic.

Schleider and colleagues conducted an RCT with 2452 adolescents (age range 13–16 years; 21.9% males) with elevated depression symptoms testing two online (self-guided) 20–30-min single session interventions (SSIs; a behavioral activation SSI and an SSI teaching that traits are malleable) with peer narratives and writing activities, compared with a supportive control [46]. Compared with the control, both active SSIs decreased post-intervention and 3-month hopelessness as measured using the Beck Hopelessness Scale. Several differences between active SSIs emerged. The authors suggested that these brief supports could be made freely available for adolescents to complete anytime, from any location, regardless of traditional barriers to mental health care.

Mesurado and colleagues measured the efficacy of a professionally guided 7-week Virtual Hero Program in a cluster-RCT to increase positive emotions and prosocial behavior of 211 Colombian adolescents (mean age, 14.1 years; 58% males) [44]. They also analyzed whether the Hero program, by directly promoting positive emotional states in adolescents, predisposed them to take prosocial actions toward other people (via an indirect or mediated effect). According to their positive emotions questionnaire and the Kindness and Generosity subscale of the Values in Action Inventory of Strengths, their results indicated that the program increased joy, gratitude, serenity, and personal satisfaction but not sympathy for those who participated in the intervention. They also found that the promotion of these positive emotions predisposed adolescents to act prosocially. The program was effective in directly promoting prosocial behaviors in adolescents during social isolation, as observed through a statistically significant difference in the pre- and post-test evaluations between the control and intervention groups. The structure of their Virtual Hero Program intervention brought adolescents closer to social situations to which isolation had limited their access, promoting the importance of closeness and solidarity with others within the complexities of the social confinement context.

Malboeuf-Hurtubise and colleagues conducted two cluster pilot and feasibility RCTs to test art therapy experientials—different activities that may be used in art therapy broadly—including an emotion-based drawing intervention and a mandala drawing intervention, in two elementary school classrooms (mean age, 11.3 years; 50% males) [42] and Philosophy for Children and a Mindfulness Intervention (in five elementary school classrooms; mean age, 8.2 years; 58% males) [43] including 22 and 37 children, respectively. Both interventions were group-based, guided by a professional and delivered online and remotely. The Behavior Assessment Scale for Children-3rd edition was used to measure outcomes for both RCTs. Analyses of covariance revealed a significant effect of the type of art therapy activities on levels of inattention, after controlling for baseline levels. Participants in the emotion-based directed drawing group showed lower inattention scores at post-test, when compared to participants in the mandala group. Post-hoc sensitivity analyses showed significant decreases in pre-to-post scores for levels of hyperactivity for the complete sample. Analyses of covariance also revealed a significant effect of the Philosophy for Children intervention on mental health difficulties, controlling for baseline levels. Participants in the Philosophy for Children group showed lower (i.e., better) scores on inattention at post-test than participants in the Mindfulness Intervention group. While future work including larger sample size and follow-up measures is warranted for all interventions from both studies, these results highlight collectively that the implementation of all interventions online and remotely, through a videoconference platform, is feasible and adequate in school-based settings.

### Learning and competence domain

Two complex RCTs included a component to address learning and competence among children and youth. These studies reported improved learning, increased positive emotions, and increased well-being [45] or decreased stress and improved sleep [48].

Sarti and colleagues conducted a professionally guided 4-month telerehabilitation program including 56 children (mean age, 10.8 years; 39% males) with special needs (specific learning disorders and cerebral palsy diagnosis) compared to children who did not undertake telerehabilitation despite the special needs diagnosis during the pandemic, and with typically developing children [45]. Using the various subscales contained within the Comprehensive Inventory for Thriving Children, they showed that the three groups differed in the Learning dimensions of well-being experience. Post hoc comparisons revealed that children with specific learning disorders and cerebral palsy scored higher than typically developing children on Respect scales. Furthermore, children who experienced telerehabilitation showed the highest scores on the Learning scale in comparison with the other two groups. The authors suggested that their results support the importance of reorganizing care and assistance by integrating telemedicine, which seems to have fostered a positive experience of well-being in children with special needs, particularly in the perception of a supportive environment that respects psychological needs.

Yadav and colleagues evaluated the use of a self-guided Heartfulness Meditation and Audio Brainwave Entrainment to help teenagers cope with mental health issues [48]. The intervention used 30-min Heartfulness meditation and 15-min brainwave entrainment sessions with binaural beats and isochronic tones three times a week for 4 weeks. The 40 participants (mean age, 16 years; 17% males) were divided into four experimental groups (control group, Audio Brainwave Entrainment group, Heartfulness Meditation group, and a combined group) and asked to complete a survey battery using pretest–posttest methodology. While the authors proved the efficacy of a 4-week Heartfulness Meditation program to regulate overall mood, stress levels, state depression, and sleep quality (on the Pittsburgh Quality of Sleep Index, Perceived Stress Scale, Patient Health Question-9, and the Profile of Mood States), using the Cambridge Brain Health Assessment, the singular Audio Brainwave Entrainment group did not see statistically significant improvements nor did any of the intervention groups for brain health, specifically memory and attention.

### Agency and resilience domain

Five included studies assessed improvements in agency and resilience among children and youth after completing their respective well-being interventions with a follow-up that ranged from 1 week to 12 months. Included interventions reported increased negative coping and increased positive coping [41], increased satisfaction and increased resilience [47], increased resilience [49], increased self-efficacy [38], and decreased hopelessness [46].

The following interventions have been described above, and only their findings on agency and resilience will be presented here. The Logotherapy-based Mindfulness Intervention by Liu and colleagues improved positive and negative coping and 121 youth as measured on the Trait Coping Style Questionnaire [41]. Shao and colleagues found that their Dance Therapy intervention increased resilience after 8 weeks (on the Measurement of Psychological Resilience) [47]. Using the Healthy Kids Resilience Assessment, Zhang and colleagues found that their 8-week research-based psychological counseling program in combination with physical exercise improved adolescents resilience [49]. Schleider and colleagues found that their SSI intervention increased agency on the Agency Subscale of the State Hope Scale among adolescents with elevated depression symptoms [46].

The effect of resilience trainings on self-efficacy of 80 elementary 9–10-year-old school girls across two schools recruited through convenience sampling in Iran was investigated by Gadari and colleagues [38]. The Children’s Social Self-Efficacy in Peer Interaction Scale was used for data collection before, immediately, and 1 month after the intervention. The self-efficacy score of students in the intervention group improved immediately and 1 month after the intervention and was significantly different than the control group. The authors concluded that resilience training may be a powerful intervention to prevent social and psychological harm in elementary school girls.

### Safety and support domain

A single study investigated the effect of a well-being intervention to improve safety and support among children and youth. This study reported increased income and decreased violence at seven months [39].

Gulesci and colleagues conducted a complex RCT including 600 vulnerable adolescents aged 15–18 recruited through convenience on a professionally guided 3-month Youth Empowerment Program that offered training in soft skills and technical skills, sexual education, mentoring, and job-finding [39]. The authors utilized yes/no answer questions via telephone follow-up to protect the safety of the vulnerable youth. Their results indicated that at a 7-month follow-up, young women (but not young men) had increased earnings and decreased violence (against them) that was measured with direct self-report questions as well as list experiments. These findings indicate that interventions aiming to empower vulnerable young women can be effective in reducing violence during periods of heightened risk.

## Discussion

We present a detailed systematic review of the effects of interventions deployed during the COVID-19 pandemic designed to improve well-being among children and youth. Although 14 of the 19 included studies (74%) provided high-quality data from RCTs, data for many interventions included small numbers or lack of real-life diversity in the sample (e.g., few studies included children under the age of 10; over 75% of included studies did not report on socioeconomic status), which limited how accurately the data represent the experiences or outcomes of all individuals or cultural groups. A minority of studies that described the development of their interventions integrated children and youth with lived experience in intervention development despite the recognition that intervention developer-user collaboration can enhance the acceptability and usefulness of innovations [52]. Owing to the rapidly changing nature of the COVID-19 pandemic, several studies deployed complex interventions (some self-guided) without prior evidence that challenged our ability to delineate effective intervention components; it is possible that any positive effect of components in aggregate may not hold at the individual level. Nonetheless, consistency in findings across studies indicates that well-being interventions for children and youth generally had positive effects. Research is needed to determine intervention modifications to support child and youth well-being during the post-pandemic phase.

Most included studies deployed mental health interventions to improve psychological well-being. Three types of interventions had the greatest supporting evidence for positive effects on child and youth well-being, specifically (1) recovery psychology (i.e., to aid in potential for recovery; e.g., Mindfulness Training [41]), (2) positive or preventive psychology (i.e., to build tools to navigate adversity; e.g., Virtual Hero Program [44]), and (3) psychology education on skills (i.e., to disseminate evidence-based recommendations for enhanced coping, treatment efficacy, and adherence; e.g., Divergent Thinking Training [51]). Effective well-being interventions often involved a digital health component (e.g., applications, internet programs, virtual reality environments) that promoted anonymity, accessibility, prompt feedback, cost-effectiveness, high treatment fidelity, and applicability in real-life contexts, which have been reported before the COVID-19 pandemic [53–56]. Considering the increased digital literacy among children and youth, evidence-based digital well-being interventions may serve as a new method to increase accessibility to well-being interventions in this priority population [57, 58].

Few studies included process evaluations to assess implementation (i.e., fidelity, dose, reach) and outcome measures (i.e., use of wrong tool) that is especially important among participants of self-guided interventions to ensure that the intervention for both control and experimental groups is received as specified in the protocol [59]. While most interventions found positive intervention effects on at least some outcomes when assessed at the last time point of follow-up, it is possible that self-guided interventions in particular were not reliably delivered as intended or consistently adhered to as required, for well-being interventions to have a full effect [60]. For example, Lee and colleagues compared the effect of synchronous online physical education classes for Tabata training with a physical education-centered asynchronous online class on the physical strength of adolescents, finding that physical fitness of adolescents was sufficiently improved only through synchronous methods. Additional work is required to uncover the differences in engagement regarding synchronous versus asynchronous interventions. We also found that it was uncommon for studies to develop interventions considering theories of long-term psychological change for children and youth (e.g., relational post-traumatic stress disorder [61]). Symptoms of distress in children and youth have been reported to develop further from the time of a traumatic experience as children and youth process their reality of experience [62]. What works in the immediate aftermath of trauma for children and youth may not be helpful months later. Future work is needed to identify and develop targeted approaches to reach sub-groups of children and youth who are most at risk of negative well-being outcomes as a consequence of COVID-19 and beyond.

We evaluated a broad scope of outcomes that were primarily measured using validated assessment tools for symptoms of mental health disorders, the most common of which being the Generalized Anxiety Disorder-7, Children’s Depression Inventory-2, Self-rating Anxiety Scale, and the Self-rating Depression Scale. In addition to assessing the improvement of negative consequences of the COVID-19 pandemic, we included four studies that explored positive adaptations [63, 64]. Hopefulness and humanity are two of many positive adaptations to changing demands of stressful experiences that is captured within one’s psychological resilience [65, 66]. Though high resiliency proactively fosters positive adaptations [67, 68], it is unknown whether positive adaptations are by-products of intervention or whether they improve coping conducts. Future work is needed to uncover the mechanisms by which well-being interventions work; well-being in children and youth should be considered holistically as the aggregate of all well-being domains.

Emerging research describes mixed evidence regarding the impact of the COVID-19 pandemic on children and youth [69]. It is reported that emotional regulation, robust resilience [70], physical activity [71], parental self-efficacy, family functioning, and social support [72] are protective factors. In contrast, exposure to excessive information [73], emotional reactivity and experiential avoidance [74], presence of COVID-19 cases in the community, COVID-19 school concerns, parental mental health problems [75], and increased Internet, social media, and video game use [76, 77] have been identified as potentially detrimental factors. Many authors of the included studies noted that what constitutes appropriate and sufficient well-being care in the context of the COVID-19 pandemic with adequate follow-up is unclear. Children and youth cope differently with stressful experiences such that any intervention that changes coping trajectories has the potential to do harm [78, 79]. We suggest that those who provide well-being interventions to children and youth should engage at a minimum children and youth as stakeholders—experts in their own rights—prior to intervention development for consideration of personal experiences to carefully weigh probable adverse effects against potential benefits [80]. Though the optimal approach to enhancing child and youth well-being during periods of health crisis is unknown, it is likely to consist of many components that may include approaches for engagement and contain customizable features based on user preference that are tailored to local settings while aimed toward all stakeholders.

### Directions for future research

Based on the results of this review, significant gaps in the existing literature on interventions to improve well-being among youth and adolescents can be discerned. Among the highest priority gaps are the need for well-designed trials that study online interventions and include appropriate and blinded control groups (e.g., active interventions or waitlists) within an adequate sample size; ethical considerations of withholding interventions in community contexts as well as the inability to blind behavioral interventions may have contributed to this knowledge gap. These interventions have been shown to be potentially effective, but confidence is limited due to lower quality and lack of replication. Independent corroborating evidence for any particular intervention is required. In addition, more structured and comprehensive analysis methodologies need to be applied. At a minimum, gender, age, and relevant social-economic variables of interest should be included as covariates. More research is needed to determine when and for whom an intervention to improve well-being would be developmentally and contextually appropriate. Capturing child and youth voices through introspective and dialogical approaches that transcend cultures is needed to inform the refinement of existing interventions to support child and youth well-being in the post-pandemic phase and for the development of preventive and responsive interventions during future health crises.

### Strengths and limitations

We used a large number of educational as well as health electronic databases independently and in duplicate assessed study eligibility, conducted data extraction, and evaluated study quality; however, we included only published literature (excluding preprints that may have increased publication bias), and it is possible that studies may have been missed. Several studies reported only descriptive analyses that did not take account of potential confounders. The studies included work performed within the first year of the COVID-19 pandemic and across 10 countries globally that represent varied and diverse pandemic experiences. More work is needed to examine the effect of well-being interventions that were tested in later waves as well as after the COVID-19 pandemic. There are likely to be long-lasting impacts of the pandemic on children and youth well-being. In addition, while several studies included participants with medical conditions (e.g., cerebral palsy), no studies were included on a number of important outcomes or vulnerable groups, including studies of children with autism or studies of eating disorders or substance use; effectiveness of interventions should be generalized with great caution to children and youth other than those in which they have been studied. Owing to the heterogeneity of included studies, it was not possible to conduct a meta-analysis; rather, the results were summarized with a narrative synthesis. We used an established conceptual framework including five broad and non-mutually exclusive domains for adolescent well-being [9]. Our broad inclusion criteria resulted in a comprehensive summary of literature assessing well-being interventions targeting children and youth during the COVID-19 pandemic; quality assessments were not performed owing to inconsistent outcome measures and assessments within each well-being domain. It was also not possible to determine specific components of interventions associated with more favorable child and youth outcomes given the diverse and multicomponent nature of interventions.

## Conclusions

In this systematic review of interventions from the first 2 years of the COVID-19 pandemic to improve child and youth well-being, studies of short-term and long-term follow-up reported improved well-being among children and youth. Data for many of these interventions came from places where small numbers or lack of real-life diversity in the sample limited how accurately the data represent the experiences or outcomes of certain individuals or specific groups. The available data indicate that future well-being interventions should be evidence-informed, data-driven, and reflective of need; developing acceptable and useful interventions to improve all aspects of youth well-being will require increased availability and access to quality data across sectors. While actions aimed at single issues are necessary and may seem simpler to implement, systemic change that upholds fundamental child rights will lead to sustainable improvement in the overall well-being of all children and youth.

## Supplementary Information

**Additional file 1:**  **Table S1** - **S4.** 

**Additional file 2:** Data abstracted from included studies.

## Data Availability

All data generated or analyzed during this study are included in this published article [and its supplementary information files].

## Abbreviations

COVID-19: Coronavirus disease 2019
SES: Socioeconomic status
PRISMA: Preferred Reporting Items for Systematic Reviews and Meta-analyses
SWiM: Synthesis Without Meta-analyses
RCT: Randomized controlled trial
CRT: Cluster randomized controlled trial
WHO: World Health Organization
USA: United States of America
