# Supplementary material for: Interventions to improve well-being among children and youth aged 6–17 years during the COVID-19 pandemic: a systematic review
Source: BMC Med. 2023 Apr 3;21:131. doi: 10.1186/s12916-023-02828-4 (PMC10069351; doi:10.1186/s12916-023-02828-4)
Supplement: Supplementary file 1 — Additional file 1: Table S1 - S4. [file 12916_2023_2828_MOESM1_ESM.docx]

**Table S1.** Preferred Reporting Items for Systematic review and Meta-Analyses (PRISMA) Checklist

| **Section/topic** | **#** | **Checklist item** | **Reported on page #** |
| --- | --- | --- | --- |
| **TITLE** | | |  |
| Title | 1 | Identify the report as a systematic review, meta-analysis, or both. | 1 |
| **ABSTRACT** | | |  |
| Structured summary | 2 | Provide a structured summary including, as applicable: background; objectives; data sources; study eligibility criteria, participants, and interventions; study appraisal and synthesis methods; results; limitations; conclusions and implications of key findings; systematic review registration number. | 3 |
| **INTRODUCTION** | | |  |
| Rationale | 3 | Describe the rationale for the review in the context of what is already known. | 4 |
| Objectives | 4 | Provide an explicit statement of questions being addressed with reference to participants, interventions, comparisons, outcomes, and study design (PICOS). | 4 |
| **METHODS** | | |  |
| Protocol and registration | 5 | Indicate if a review protocol exists, if and where it can be accessed (e.g., Web address), and, if available, provide registration information including registration number. | 4 |
| Eligibility criteria | 6 | Specify study characteristics (e.g., PICOS, length of follow-up) and report characteristics (e.g., years considered, language, publication status) used as criteria for eligibility, giving rationale. | 5 |
| Information sources | 7 | Describe all information sources (e.g., databases with dates of coverage, contact with study authors to identify additional studies) in the search and date last searched. | 5 |
| Search | 8 | Present full electronic search strategy for at least one database, including any limits used, such that it could be repeated. | 5 |
| Study selection | 9 | State the process for selecting studies (i.e., screening, eligibility, included in systematic review, and, if applicable, included in the meta-analysis). | 5 |
| Data collection process | 10 | Describe method of data extraction from reports (e.g., piloted forms, independently, in duplicate) and any processes for obtaining and confirming data from investigators. | 6 |
| Data items | 11 | List and define all variables for which data were sought (e.g., PICOS) and any assumptions and simplifications made. | 6 |
| Risk of bias in individual studies | 12 | Describe methods used for assessing risk of bias of individual studies (including specification of whether this was done at the study or outcome level), and how this information is to be used in any data synthesis. | 6 |
| Summary measures | 13 | State the principal summary measures (e.g., risk ratio, difference in means). | N/A |
| Synthesis of results | 14 | Describe the methods of handling data and combining results of studies, if done, including measures of consistency (e.g., I^2^) for each meta-analysis. | 6 |

| **Section/topic** | **#** | **Checklist item** | **Reported on page #** |
| --- | --- | --- | --- |
| Risk of bias across studies | 15 | Specify any assessment of risk of bias that may affect the cumulative evidence (e.g., publication bias, selective reporting within studies). | N/A |
| Additional analyses | 16 | Describe methods of additional analyses (e.g., sensitivity or subgroup analyses, meta-regression), if done, indicating which were pre-specified. | N/A |
| **RESULTS** | | |  |
| Study selection | 17 | Give numbers of studies screened, assessed for eligibility, and included in the review, with reasons for exclusions at each stage, ideally with a flow diagram. | 6 |
| Study characteristics | 18 | For each study, present characteristics for which data were extracted (e.g., study size, PICOS, follow-up period) and provide the citations. | 6 |
| Risk of bias within studies | 19 | Present data on risk of bias of each study and, if available, any outcome level assessment (see item 12). | 6 |
| Results of individual studies | 20 | For all outcomes considered (benefits or harms), present, for each study: (a) simple summary data for each intervention group (b) effect estimates and confidence intervals, ideally with a forest plot. | 7-12 |
| Synthesis of results | 21 | Present results of each meta-analysis done, including confidence intervals and measures of consistency. | N/A |
| Risk of bias across studies | 22 | Present results of any assessment of risk of bias across studies (see Item 15). | N/A |
| Additional analysis | 23 | Give results of additional analyses, if done (e.g., sensitivity or subgroup analyses, meta-regression [see Item 16]). | N/A |
| **DISCUSSION** | | |  |
| Summary of evidence | 24 | Summarize the main findings including the strength of evidence for each main outcome; consider their relevance to key groups (e.g., healthcare providers, users, and policy makers). | 12-14 |
| Limitations | 25 | Discuss limitations at study and outcome level (e.g., risk of bias), and at review-level (e.g., incomplete retrieval of identified research, reporting bias). | 14 |
| Conclusions | 26 | Provide a general interpretation of the results in the context of other evidence, and implications for future research. | 15 |
| **FUNDING** | | |  |
| Funding | 27 | Describe sources of funding for the systematic review and other support (e.g., supply of data); role of funders for the systematic review. | 2 |

Moher D, Liberati A, Tetzlaff J, Altman DG, The PRISMA Group (2009). Preferred Reporting Items for Systematic Reviews and Meta-Analyses: The PRISMA Statement. PLoS Med 6.

**Table S2.** Prefer Synthesis Without Meta-analysis (SWiM) Reporting Items

| **SWiM is intended to complement and be used as an extension to PRISMA** | | |
| --- | --- | --- |
| **Reporting item** | **Item description** | **Reported on Page #** |
| *Methods* | | |
| **1** Grouping studies for synthesis | 1a) Provide a description of, and rationale for, the groups used in the synthesis (e.g., groupings of populations, interventions, outcomes, study design) | 8 |
|  | 1b) Detail and provide rationale for any changes made subsequent to the protocol in the groups used in the synthesis | 5 |
| **2** Describe the standardised metric and transformation methods used | Describe the standardised metric for each outcome. Explain why the metric(s) was chosen, and describe any methods used to transform the intervention effects, as reported in the study, to the standardised metric, citing any methodological guidance consulted | 8 |
| **3** Describe the synthesis methods | Describe and justify the methods used to synthesise the effects for each outcome when it was not possible to undertake a meta-analysis of effect estimates | 8 |
| **4** Criteria used to prioritise results for summary and synthesis | Where applicable, provide the criteria used, with supporting justification, to select the particular studies, or a particular study, for the main synthesis or to draw conclusions from the synthesis (e.g., based on study design, risk of bias assessments, directness in relation to the review question) | 8 |
| **Reporting item** | **Item description** | **Reported on Page #** |
| **5** Investigation of heterogeneity in reported effects | State the method(s) used to examine heterogeneity in reported effects when it was not possible to undertake a meta-analysis of effect estimates and its extensions to investigate heterogeneity | 8 |
| **6** Certainty of evidence | Describe the methods used to assess certainty of the synthesis findings | 8 |
| **7** Data presentation methods | Describe the graphical and tabular methods used to present the effects (e.g., tables, forest plots, harvest plots).  Specify key study characteristics (e.g., study design, risk of bias) used to order the studies, in the text and any tables or graphs, clearly referencing the studies included | 9-17 |
| *Results* | | |
| **8** Reporting results | For each comparison and outcome, provide a description of the synthesised findings, and the certainty of the findings. Describe the result in language that is consistent with the question the synthesis addresses, and indicate which studies contribute to the synthesis | 9-17 |
| *Discussion* |  |  |
| **9** Limitations of the synthesis | Report the limitations of the synthesis methods used and/or the groupings used in the synthesis, and how these affect the conclusions that can be drawn in relation to the original review question | 21 |

Campbell M, McKenzie JE, Sowden A, Katikireddi SV, Brennan SE, Ellis S, Hartmann-Boyce J, Ryan R, Shepperd S, Thomas J, Welch V, Thomson H. Synthesis without meta analysis (SWiM) in systematic reviews: reporting guideline. BMJ 2020;368:l6890.

**Table S3.** Complete MEDLINE search strategy

| **Setting** | **Population** | **Design** | **All** |
| --- | --- | --- | --- |
| 1 COVID-19/ or SARS-CoV-2/  2 (coronavirus/ or betacoronavirus/ or coronavirus infections/) and (disease outbreaks/ or epidemics/ or pandemics/)  3 (nCoV* or 2019nCoV or 19nCoV or COVID19* or COVID or SARS-COV-2 or SARSCOV-2 or SARS-COV2 or SARSCOV2 or SARS coronavirus 2 or Severe Acute Respiratory Syndrome Coronavirus 2 or Severe Acute Respiratory Syndrome Corona Virus 2).tw,kf.  4 ((new or novel or "19" or "2019" or Wuhan or Hubei or China or Chinese) adj3 (coronavirus* or corona virus* or betacoronavirus* or CoV or HCoV)).tw,kf.  5 (long COVID* or longCOVID* or postCOVID* or post-COVID* or postcoronavirus* or post-coronavirus* or postSARS* or post-SARS*).tw,kf.  6 ((coronavirus* or corona virus* or betacoronavirus*) adj3 (pandemic* or epidemic* or outbreak* or crisis)).tw,kf.  7 ((Wuhan or Hubei) adj5 pneumonia).tw,kf.  8 1 or 2 or 3 or 4 or 5 or 6 or 7  9 limit 8 to yr="2019 -Current"  10 animals/ not humans/  11 9 not 10 | 12 adolescent/ or child/  13 (adolescen* or child or children or teen* or youth*).tw,kf.  14 12 or 13  15 11 and 14  16 limit 11 to ("child (6 to 12 years)" or "adolescent (13 to 18 years)")  17 15 or 16 | 18 ((randomized controlled trial or controlled clinical trial).pt. or randomized.ab. or randomised.ab. or placebo.ab. or drug therapy.fs. or randomly.ab. or trial.ab. or groups.ab.) not (exp animals/ not humans.sh.) | 19 17 and 18 |

**Table S4.** Risk of bias assessment for all included studies

|  | **Selection bias** | | **Performance bias** | **Detection bias** | **Attrition bias** | **Reporting bias** | **Overall Risk for Bias** |
| --- | --- | --- | --- | --- | --- | --- | --- |
| **Study** | **Random sequence generation^1^** | **Allocation concealment** | **Blinding of participants and researchers** | **Blinding of outcome assessment** | **Incomplete outcome data^1^** | **Selective reporting** |  |
| Cataldi | Low | Low | Low | Low | Low | Low | Low |
| Chen | Unclear | Unclear | High | Low | Low | Low | High |
| Choi | Low | Low | Low | Low | Low | Low | Low |
| Cruwys | Low | Low | Low | Low | Low | Low | Low |
| Ding | Unclear | Unclear | High | Low | Low | Low | High |
| Gadari | Unclear | Unclear | High | Low | Low | Low | High |
| Gulesci | Low | Low | Low | Low | Low | Low | Low |
| Lee | Low | Low | Low | Low | Low | Low | Low |
| Liu | Unclear | Unclear | High | Low | Low | Low | High |
| Malboeuf-Hurtubise (a) | Low | Low | Low | Low | Low | Low | Low |
| Malboeuf-Hurtubise (b) | Low | Low | Low | Low | Low | Low | Low |
| Mesurado | Unclear | Low | High | Low | High | High | High |
| Sarti | Unclear | Unclear | High | Unclear | Unclear | Low | High |
| Schleider | Low | Low | Low | Low | Low | Low | Low |
| Shao | Low | Unclear | High | High | Unclear | Unclear | High |
| Yadav | Unclear | Unclear | Low | Low | Low | Low | Unclear |
| Zhang | Unclear | Low | Low | Low | Low | Low | Unclear |
| Zheng | Low | Low | Low | Low | High | High | High |
| Zuo | Low | Low | Low | Low | Low | Low | Low |

Determined by the Cochrane Risk of Bias Assessment Tool; NB. Nearly all studies were unblinded to participants and researchers.

Study did not receive a score if no control group was included

^1^Overall attrition above 20% represents high risk of attrition bias; ratings of unclear represent that either overall attrition or attrition between groups was not reported
